# Association Between State Policies Using Medicaid Exclusions to Sanction Noncompliance With Welfare Work Requirements and Medicaid Participation Among Low-Income Adults

**DOI:** 10.1001/jamanetworkopen.2020.4579

**Published:** 2020-05-11

**Authors:** Atheendar S. Venkataramani, Elizabeth F. Bair, Erica Dixon, Kristin A. Linn, William J. Ferrell, Kevin G. Volpp, Kristen Underhill

**Affiliations:** 1 Department of Medical Ethics and Health Policy, Perelman School of Medicine, University of Pennsylvania, Philadelphia; 2 Department of Medicine, Perelman School of Medicine, University of Pennsylvania, Philadelphia; 3 Center for Health Incentives and Behavioral Economics, University of Pennsylvania, Philadelphia; 4 Department of Biostatistics, Epidemiology, and Informatics, Perelman School of Medicine, University of Pennsylvania, Philadelphia; 5 Columbia Law School, New York, New York; 6 Mailman School of Public Heath, Department of Population and Family Health, Columbia University, New York, New York

## Abstract

This cohort study examines the association of implementation of Medicaid sanctions in the Temporary Assistance for Needy Families program with Medicaid coverage rates among low-income adults.

## Introduction

Twenty states have pursued community engagement requirements (ie, work requirements) as a condition for Medicaid eligibility among adults considered able-bodied. Work requirements seek to improve health by incentivizing work,^1^ but may result in coverage losses.^2^

The impact of work requirements on Medicaid coverage may extend beyond qualifying beneficiaries, by increasing confusion around benefit rules or deterring individuals from applying for coverage.^3^ However, the spillover effects of work requirements on individuals not directly subject to them are difficult to study because these programs have only recently been implemented. To examine this possibility, we studied Temporary Assistance for Needy Families (TANF), the cash welfare program enacted under welfare reform in 1996. The TANF program requires able-bodied beneficiaries to fulfill work requirements, and states can elect to terminate Medicaid benefits as a sanction for nonpregnant adult TANF participants who do not comply with them.^4^ In states adopting these sanctions (Table), Medicaid eligibility for dual TANF-Medicaid enrollees was effectively conditional on meeting work requirements. This quasi-experimental cohort study examines whether TANF-Medicaid sanctions had spillover effects on Medicaid coverage among low-income adults who were not likely to participate in TANF and, therefore, were not directly subject to these sanctions.

**Table.** Selected Descriptive Statistics^a^

Characteristic	Residents, No. (weighted %)	
	States with TANF sanctions^b^	States without TANF sanctions
Total	61 499 (19.1)	227 713 (80.9)
Individual characteristics		
Age, mean (SD), y	34.5 (9.8)	34.4 (9.8)
Sex		
Female	24 843 (40.9)	94 451 (43.0)
Male	36 656 (59.1)	133 266 (57.0)
Race/ethnicity		
Non-Hispanic white	45 234 (67.9)	176 490 (74.8)
Black	13 499 (29.0)	34 139 (18.9)
Hispanic	2407 (2.8)	14 509 (5.6)
Other	357 (0.3)	2579 (0.8)
Education level		
High school or less	42 351 (68.9)	158 775 (69.7)
Some college or more	19 148 (31.1)	68 942 (30.3)
Married	26 834 (43.6)	101 226 (44.4)
State characteristics, %		
Unemployment rates	5.6	5.7
Poverty rate	14.2	13.6
Implemented TANF family sanction^c^	45.1	19.5

Abbreviations: TANF, Temporary Assistance for Needy Families.

^a^ All descriptive statistics were weighted by Current Population Survey sampling weights. The primary analytic sample consisted of adults aged 18 to 55 years living in households reporting annual incomes at or below 185% of the federal poverty line interviewed over the period 1991 to 2003. Sample excludes single mothers, who were most likely to receive welfare benefits and were thus most affected by welfare reform efforts.

^b^ States with TANF Medicaid sanctions in place as of 2000 were Alabama, Idaho, Indiana, Kansas, Louisiana, Michigan, Mississippi, Nebraska, New Mexico, Nevada, Ohio, South Carolina, and Wyoming. The sanctions allowed termination of Medicaid benefits for nonpregnant household heads receiving TANF benefits who did not meet TANF work requirements.

^c^ These sanctions allowed termination of TANF benefits for family members of individuals who did not meet TANF work requirements.

## Methods

In accordance with University of Pennsylvania guidelines, institutional review board approval and informed consent were not required for this study because the data were publicly available and deidentified. This study follows the Strengthening the Reporting of Observational Studies in Epidemiology (STROBE) reporting guideline.

We used individual-level data from the 1991 to 2003 Annual Social and Economic Supplement of the Current Population Survey, encompassing the 10 years surrounding TANF adoption (1996-1998), combined with state-level information on TANF-Medicaid sanctions in place as of 2000.^4^ We restricted our sample to adults aged 18 to 55 years living in households with incomes less than 185% of the federal poverty level in order to capture adults most likely to participate in Medicaid and other means-tested benefit programs. To focus specifically on spillover effects, we excluded single mothers, the population most likely to access TANF.^5^

We estimated difference-in-differences models comparing changes in Medicaid participation among individuals living in states with TANF-Medicaid sanctions in each 2-year period before and after implementation with similar changes among individuals living in states without sanctions. We adjusted for individual demographic and socioeconomic characteristics, state-year level policy and economic factors (ie, timing of TANF adoption, adoption of full-family sanctions for TANF noncompliance, adult Medicaid income eligibility thresholds, unemployment rates, and poverty rates), and state and year fixed effects.^4,6^ The 95% CIs were adjusted for clustering at the state level. Analyses used Stata statistical software version 16.1 (StataCorp). All *P* values were from 2-sided *t* tests, with statistical significance set at *P* < .05. Data analysis was performed from February 2018 to March 2020.

## Results

Our sample included 289 216 adults (mean [SD] age, 33.3 [10.3] years; 169 922 men [weighted 59%]); 61 499 (weighted 19.1%) resided in states implementing TANF-Medicaid sanctions during the study period (Table). Most participants were non-Hispanic white (221 724 participants [77%]) and most had a high school education or less (201 126 participants [70%]).

The Figure plots difference-in-difference estimates. We found no visual or statistical evidence of differential preimplementation trends in Medicaid participation for sanction-adopting vs nonadopting states (*b* = 0.0028; 95% CI, −0.0027 to 0.0083; *P* = .32). After implementation, Medicaid participation declined in sanction-adopting states compared with nonadopting states. On average, across all postimplementation years, adoption of TANF-Medicaid sanctions was associated with an absolute reduction in Medicaid coverage of 2.4 percentage points (95% CI, −4.6 to −0.16 percentage points; *P* = .04), representing a 10.1% relative decline from the preimplementation Medicaid participation rate (23.6%). In a secondary analysis, we found that associations with overall insurance coverage were not statistically significant (1.2 percentage point decrease; 95% CI, −3.7 to 1.2 percentage points; *P* = .32).

**Figure.**
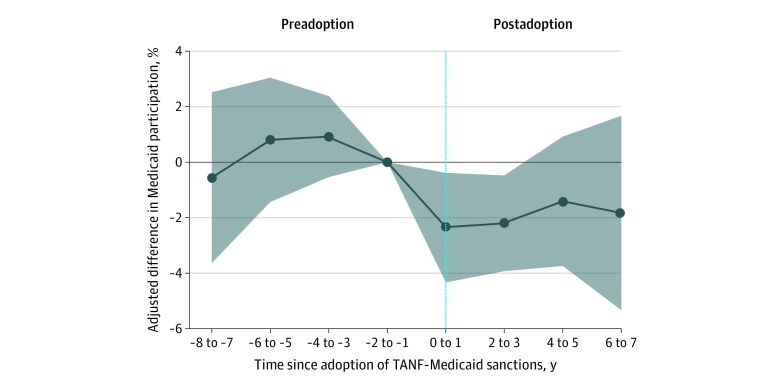
Difference-in-Differences Estimates of Association Between Implementation of Medicaid Sanctions in Temporary Assistance for Needy Families (TANF) Program and Medicaid Coverage Estimates were generated from linear probability (ordinary least squares) difference-in-differences models. The exposure of interest was a binary indicator of adoption of TANF Medicaid sanctions. All models were adjusted for fixed effects for respondent age (years), sex, race/ethnicity, education (in years), and marital status; state-year measures of TANF adoption (binary indicator); implementation of TANF sanctions for family members (binary indicator), poverty rates (percentage), unemployment rates (percentage), and Medicaid eligibility thresholds (percentage federal poverty line); and state and year fixed effects (to adjust for time invariant state-level confounders and national secular trends). Each point represents the difference-in-difference coefficient for a specified 2-year event period on the x-axis, with the 2-year period before policy adoption serving as the reference group. The 95% CIs (shaded area) were adjusted for clustering at the state (policy) level (no CI was estimated for the period −2 to −1 years, given this was the reference group).

## Discussion

Medicaid sanctions for noncompliance with TANF work requirements were associated with decreased Medicaid participation among low-income individuals not subject to these sanctions. Study limitations include possible bias from unobserved confounders, attenuation bias from potential measurement error (eg, incomplete designation of sanction-adopting states), and unknown generalizability to modern programs.

Nevertheless, our findings raise the possibility that Medicaid work requirements may lead to reduced program participation even among individuals who are exempt from the obligations. Our estimate of the spillover effect—a relative decline of 10.1%—is more than one-half as large as the direct effect of Arkansas’ now terminated Medicaid work requirement on insurance coverage (18% relative decline).^2^ The spillover consequences of work requirements may thus be substantial.

## References

[1] Verma S Remarks by administrator Seema Verma at the 2018 Medicaid managed care summit. Published September 27, 2018 Accessed February 12, 2018. https://www.cms.gov/newsroom/press-releases/speech-remarks-administrator-seema-verma-2018-medicaid-managed-care-summit

[2] Sommers BD, Goldman AL, Blendon RJ, Orav EJ, Epstein AM Medicaid work requirements: results from the first year in Arkansas. N Engl J Med. 2019;381(11):1073-1082. doi:10.1056/NEJMsr1901772 31216419

[3] Chavkin W, Romero D, Wise PH State welfare reform policies and declines in health insurance. Am J Public Health. 2000;90(6):900-908. doi:10.2105/AJPH.90.6.900 10846507 PMC1446262

[4] US General Accounting Office Welfare Reform: State Sanction Policies and Number of Families Affected. US General Accounting Office; 2000.

[5] Kaestner R, Kaushal N Welfare reform and health insurance coverage of low-income families. J Health Econ. 2003;22(6):959-981. doi:10.1016/j.jhealeco.2003.06.004 14604555

[6] University of Kentucky Center for Poverty Research UKCPR national welfare data, 1980-2017. Published 2019 Accessed August 13, 2019. http://ukcpr.org/resources/national-welfare-data

